# Author Correction: A political economy theory of fossil fuel subsidy reforms in OECD countries

**DOI:** 10.1038/s41467-024-51013-5

**Published:** 2024-08-01

**Authors:** Nils Droste, Benjamin Chatterton, Jakob Skovgaard

**Affiliations:** 1 Department of Political Science and Centre for Innovation Research, Lund University, Box 117, 221 00 Lund, Sweden; 2 Department of Economic History, Lund University, Box 117, 221 00 Lund, Sweden; 3 Department of Political Science, Lund University, Box 117, 221 00 Lund, Sweden

Correction to: *Nature Communications* 10.1038/s41467-024-49835-4, published online 27 June 2024

The original version of this Article contained an error in Fig. 2, in which the arrow from “fossil fuel subsidy levels” should have pointed back to “renewables’ market shares” directly.

This has been corrected in both the PDF and HTML versions of the Article.

