# Supplementary material for: A political economy theory of fossil fuel subsidy reforms in OECD countries
Source: Nat Commun. 2024 Jun 27;15:5452. doi: 10.1038/s41467-024-49835-4 (PMC11211386; doi:10.1038/s41467-024-49835-4)
Supplement: Supplementary file 1 — Supplementary Information [file 41467_2024_49835_MOESM1_ESM.pdf]

Supplementary Information for “A political economy theory of fossil fuel subsidy reforms in OECD countries”

Nils Droste[1]*, Benjamin Chatterton[2], Jakob Skovgaard[3]

2024

[1] Department of Political Science and Centre for Innovation Research, Lund University, Box 117, 221 00 Lund, Sweden,
[*]corresponding author, nils.droste@svet.lu.se, ORCID: 0000-0003-4357-9115
[2] Department of Economic History, Lund University, Box 117, 221 00 Lund, Sweden, ORCID: 0009-0004-2885-2181
[3] Department of Political Science, Lund University, Box 117, 221 00 Lund, Sweden, ORCID: 0000-0002-8814-2534

Supplementary Discussion

Supplementary Discussion 1: Temporal patterns

The temporal development of our main variables of interest is outlined in Supplementary Figure 1. While the cumulative number of reforms is increasing, the yearly number of reforms peaked in 2011 and started to decline after that year. Total subsidy levels peaked around 2012 (panel b, Fig. A1), decreased after that, and have fluctuated around $110-120 per capita since 2014 (with the drop in 2020 probably to the Covid pandemic). Overall, there is an average decrease from 2010 to 2020. Production-based CO2 emissions have continued to decline with more substantial drops during global financial crisis in 2008 and the pandemic in 2020. Thus, fossil fuels subsidy reforms generally increase, fossil fuels subsidy levels and production-based CO2 emissions (as opposed to consumption-based emissions) generally decrease. While these developments thus generally point into the right direction, the speed is insufficient for meeting the 1.5 target or phasing out fossil fuel subsidies by 2050.

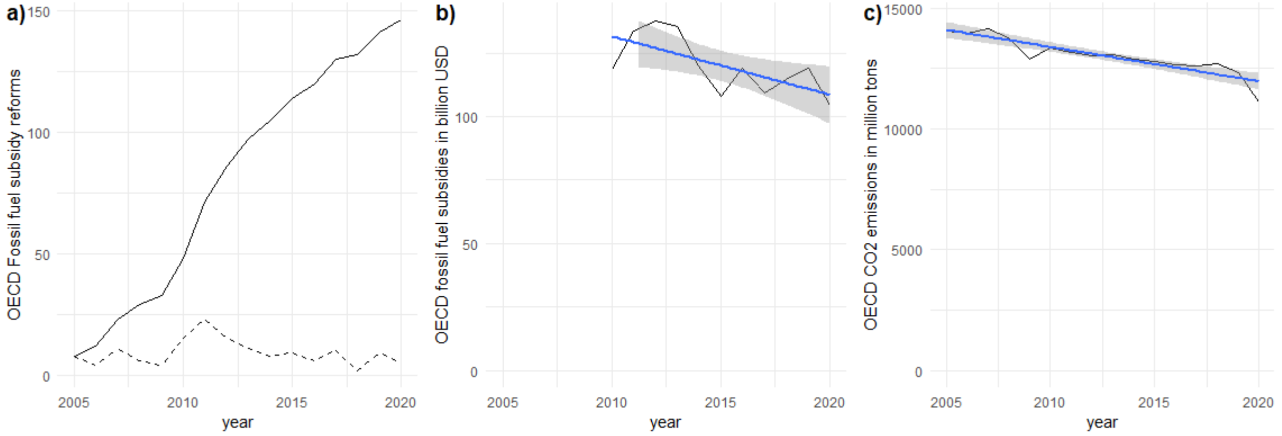

Supplementary Figure 1: Temporal development of a) the aggregate number of fossil fuel subsidy reforms (bold black line in panel a) and yearly additional reforms (dashed black line in panel a), b) the development fossil fuel subsidies (black line panel b) and estimated linear trend (blue with grey standard error range panel b), and c) the production-based CO2 emissions (black line panel c) and estimated linear trend (blue with grey standard error range panel c).

Supplementary Discussion 2: Spatial patterns

Organisation for Economic Co-operation and Development (OECD) countries vary with regard to subsidy forms, levels, and emissions. Sweden and France have the highest average number of yearly reforms over a 2005-2020 period, whereas New Zealand and Switzerland have an average of zero (see database). Ireland, Switzerland, and Australia have the highest subsidy levels per capita and New Zealand and the Netherlands the lowest. The patterns are not clear and warrant a more in-depth empirical analysis, as for instance, Switzerland has one of the highest per capita fossil fuel subsidy levels and the lowest number of reforms, while it is the opposite for New Zealand. To develop a better understanding what factors matter for fossil fuel subsidy levels and reforms, we conducted an exploratory and inductive empirical analysis, which we subsequently used to develop the theoretical explanations about the political economy of fossil fuel subsidy reforms included in the manuscript.

Supplementary Discussion 3: Inductive analysis of patterns for causal hypothesis generation

When it comes to the number of reforms, regression trees reveal an important temporal development: the number of reforms have increased over time, and the dynamics have changed over time. Pre-2012, there were less than one reform per country per year, and relatively higher number of reforms were associated with a higher renewable energy share (i.e. over 24%) and a higher CO_2 intensity (i.e. over 2.2). Post-2012, the average number of reforms per country-year observation increased to 3.4. This corroborates that there is a general time trend towards more reforms (Supplementary Figure 2) and it is possible to identify factors that separate observations with higher numbers of reforms from observations with lower numbers. Of these factors, specific economic and institutional factors stand out. Important economic (including market power) factors mainly include the structure of energy production in terms of renewable energy consumption as a percent of total energy consumption, CO_2 intensity, and CO_2 emissions and GDP per capita. The institutional factors cover the total amount of fossil fuel subsidies (due to the path-dependencies they create), state-owned oil companies, as well as executive and legislative corruption. Concerning economic factors, the analysis shows that

observations with renewable energy share were more inclined to reform their subsidies. Concerning institutional factors, post-2012 higher levels of subsidies as a share of GDP correspond to higher levels of subsidy reforms. State ownership of oil companies and legislative and executive corruption similarly correlate with more reforms for small sub-sets of the observations that have specific characteristics (e.g. low subsidies per capita, post-2012 in the case of state-owned oil companies).

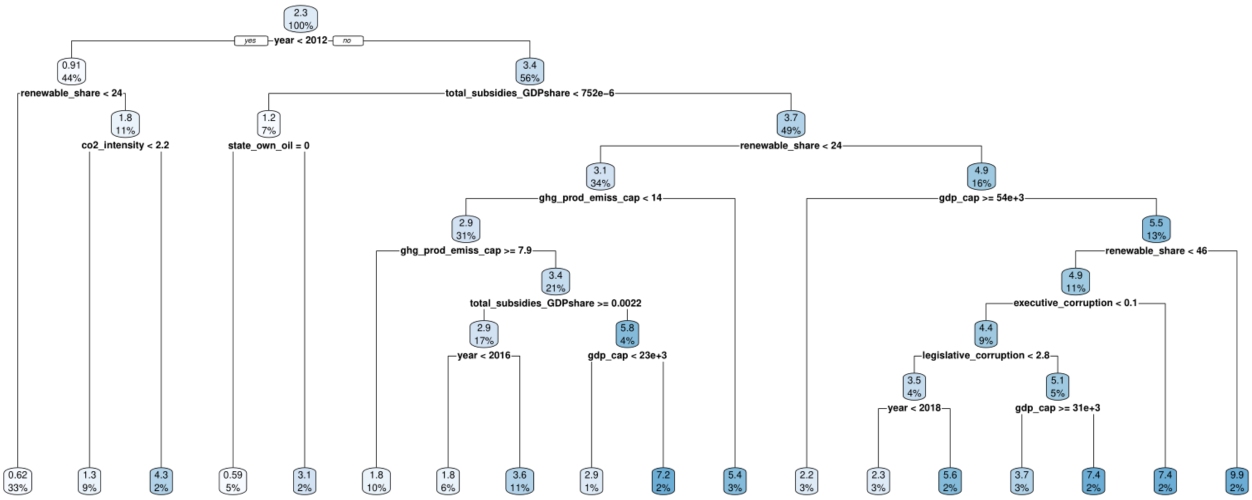

Supplementary Figure 2: Regression tree on the number of fossil fuel subsidy reforms. The tree explains the patterns by subsequently splitting the country-year observations into partitions in order of their explanatory power for the variation in the outcome variable at cut-off values of explanatory variables. There is an average value of 2.3 reforms in the whole data set (node 0 at the top of the tree covers 100 per cent of observations). The first split divides the data temporally into two partitions at a cut of value of before and from 2012. The left hand partition has an average value of 0.91 reforms (for 44% of the observations), and the right hand partition has an average value of 3.4 reforms for 56% of the data. The regression tree continues to split the data until the complexity parameter tells the tree to go no further (maximizing predictive power). Here, we present a pruned simple but not further optimized tree.

Regarding the subsidy levels, time plays less of a role. When measured in terms of fossil fuel subsidies per capita, the economic variable of GDP per capita is the most notable one, with countries with high per capita income having subsidy levels that are more twice as high as relatively poorer countries (Supplementary Figure 3). The factors correlating with higher subsidy levels are different in the former group than in the latter. Economic variables (CO_2 emissions and GDP per capita) are in general important to subsidies per capita. Institutional variables, most notably government effectiveness but also the quality of democracy and public corruption also figure prominently. While observations with low government effectiveness or high corruption have higher subsidies per capita, among both poorer and richer countries, the role of the other institutional variables are to a larger degree conditional on other variables such as CO_2 emissions, government effectiveness or corruption itself.

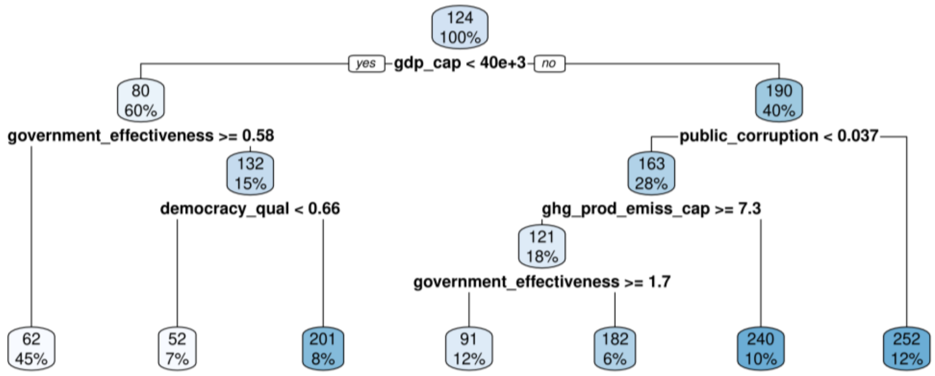

Supplementary Figure 3: Regression tree on the amount of fossil fuel subsidies (FFS) per capita. The tree explains the patterns by subsequently splitting the country-year observations into partitions in order of their explanatory power for the variation in the outcome variable at cut-off values of explanatory variables. There is an average value of 124 international dollars FFS per capita in the whole data set (node 0 at the top of the tree covers 100 per cent of observations). The first split divides the data into two partitions at a cut off value of a GDP capita of i$ 40.000. The left hand partition has an average value of i$ 80 FFS (for 60% of the observations), and the right hand partition has an average value of 190 FFS for 40% of the data. The regression tree continues to split the data until the complexity parameter tells the tree to go no further (maximizing predictive power). Here, we present a pruned simple but not further optimized tree.

Given the importance of GDP to fossil fuel subsidy levels, it is worth focusing on the level of subsidies per unit of GDP (Supplementary Fig 4). Here, we find that government effectiveness plays the most prominent role, with observations with low government effectiveness having subsidies per GDP more than are three times as large as observations with higher government effectiveness. Furthermore, the roles of other variables differ between observations with low and high effectiveness. For instance, a higher share of renewable energy comes with higher fossil fuel subsidies in percentage of GDP, within the higher government effectiveness branch and conditional on higher public corruption. Other institutional variables also play a role, including public corruption and quality of democracy, the former perhaps surprisingly correlating with high levels of subsidies per GDP for a sub-set of cases. Considering that the dependent variable includes GDP per capita, it is not surprising that economic variables play smaller, more conditional roles.

Looking at the number of reforms and subsidy levels together, a few things become apparent. First, that institutions matter. In general, countries with higher quality of political institutions (especially government effectiveness) tend to have lower levels of subsidies, whereas the direction of the relationship is less clear-cut when it comes to the number of reforms. The former finding is in line with e.g. Lockwood[1] and Victor[2], who have argued that fossil fuel subsidies are often adopted in countries with low governance capacity, partly because they constitute a way of providing welfare, and partly because fossil fuel subsidies create their own constituencies that fight to maintain and increase fossil fuel subsidies, and are more successful in countries with higher levels of clientelism. It is important that these dynamics also play out among the countries in the data set, which generally are characterized by stronger institutions than the developing countries in which these dynamics have been identified[1,3]. The fact that institutions matter may not come to a surprise to political scientists and experts on fossil fuel subsidies in developing countries.[4] Yet, the importance of such institutions also among OECD countries is highly relevant and underscores that similar dynamics shape fossil fuel subsidies both among industrialized and developing countries. On the other hand, the importance of political institutions and how other variables are conditional upon them demonstrates the importance of political context and why one-size-fits-all approaches to fossil fuel subsidy reform may fail.

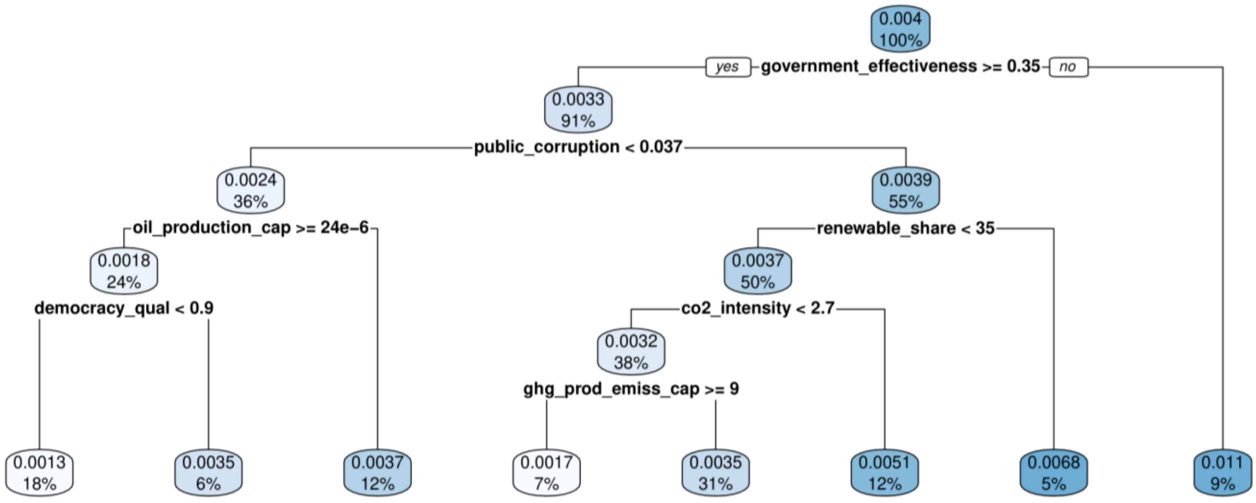

Supplementary Figure 4: Regression tree on the amount of fossil fuel subsidies (FFS) as a share of GDP in per cent. The tree explains the patterns by subsequently splitting the country-year observations into partitions in order of their explanatory power for the variation in the outcome variable at cut-off values of explanatory variables. There is an average value of 0.004 percent of GDP being spend on FFS in the whole data set (node 0 at the top of the tree covers 100 per cent of observations). The first split divides the data into two partitions at a cut off value 0.35 for the government effectiveness indicator from WDI. The left hand partition has an average value of 0.0033 FFS in per cent of GDP (for 91% of the observations), and the right hand partition has an average value of 0.011 FFS as percent of GDP for 9% of the data. The regression tree continues to split the data until the complexity parameter tells the tree to go no further (maximizing predictive power). Here, we present a pruned simple but not further optimized tree.

Second, the relationship between the number of reforms and subsidy levels is complex. While the level of subsidies plays a role for the number of reforms (at least after 2011), it is not evident from the regression tree analysis if the number of reforms plays a role for subsidy levels. It is worth speculating about the causal mechanisms behind these relationships. For instance, it appears that a higher subsidy level leads to a higher likelihood of reform, which could be due to these countries having more

subsidies that can be reformed, but also that higher expenditure on these subsidies leads to increased pressure to reform them. The absence of a clear link between the number of reforms and subsidy levels indicate that reform matters little for subsidy levels, possibly because it is only a small share of the reforms that are reformed, or because it is the smaller, less politically entrenched subsidies that are selected for reform. The recent slowdown in the number of reforms per year could reflect the latter dynamic: the lowest hanging fruits (easiest to reform) were picked first, and once this had happened; fossil fuel subsidy reform became more difficult.

To give an insight into the explanatory power of different variables, Supplementary Figure 5 indicates the variable importance for three different dependent variables, based on bagged regression trees (see Methods).

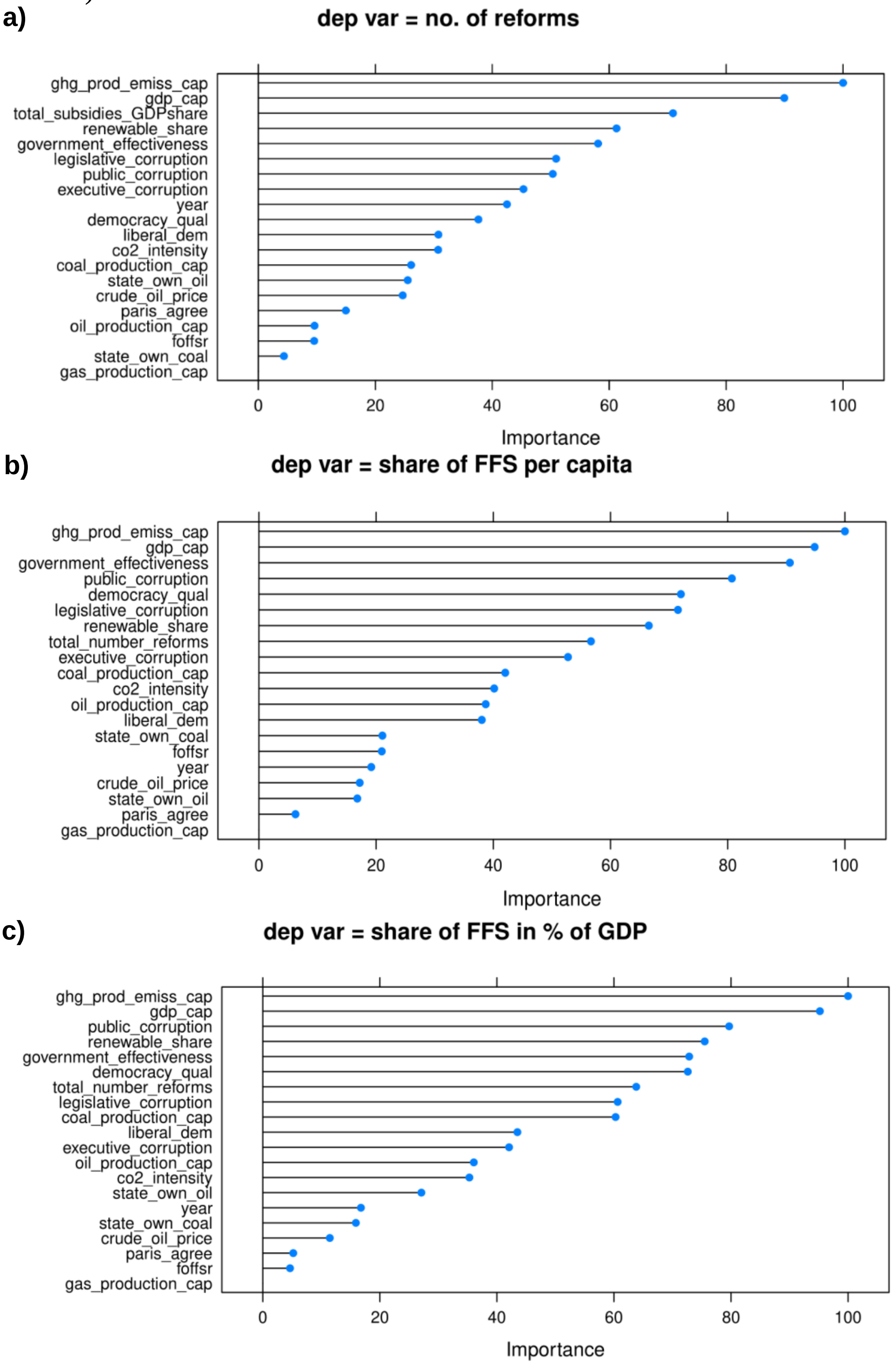

Supplementary Figure 5: Variable importance for different dependent variables. The panels indicate the share of models that included the explanatory variables in a bootstrapped set of 1000 bagged regression trees for the following dependent variables: a) the total number of reforms, b) the share of fossil fuel subsidies per capita, and c) the share of fossil fuel subsidies in per cent of gross domestic product.

Supplementary References

1. Lockwood, M. Fossil Fuel Subsidy Reform, Rent Management and Political Fragmentation in Developing Countries. *New Polit. Econ.* **20**, 475–494 (2015).
2. Victor, D. *The Politics of Fossil-Fuel Subsidies*. https://www.iisd.org/publications/report/untold-billions-fossil-fuel-subsidies-their-impacts-and-path-reform-summary-key (2009).
3. Inchauste, G. & Victor, D. G. *The political economy of energy subsidy reform*. (World Bank Publications, 2017).
4. Golden, M. A. & Mahdavi, P. The institutional components of political corruption. *Routledge Handb. Comp. Polit. Inst.* 404–420 (2015).
