# Supplementary material for: A political economy theory of fossil fuel subsidy reforms in OECD countries
Source: Nat Commun. 2024 Jun 27;15:5452. doi: 10.1038/s41467-024-49835-4 (PMC11211386; doi:10.1038/s41467-024-49835-4)
Supplement: Supplementary file 3 — Description of Additional Supplementary Files [file 41467_2024_49835_MOESM3_ESM.pdf]

Description of Additional Supplementary Files

Supplementary Data Legend

Supplementary Data 1: This file contains marginal effects for estimates of total number of reforms at different levels of government effectiveness and corruption control for all countries and years 2010, 2015, 2019, ceteris paribus.
